# Inspiratory Muscle Training Before Total Joint Arthroplasty to Improve Postoperative Outcomes: Protocol for a Randomized Controlled Trial

**DOI:** 10.2196/103290

**Published:** 2026-09-11

**Authors:** Barbara K Smith, Julie Shaner, Adam Gitlin, William Meier, Sabrina Wang, Maribel Ciampitti, Tatiana Elias-Grajeda, Ushna Khan, Johnny Washington, Michael Freidl, Stefan Braunecker

**Affiliations:** 1 Department of Physical Therapy, University of Florida, 1225 Centre Drive, Gainesville, FL, 32610, United States, 1 352 273 6855; 2 Department of Orthopaedic Surgery and Rehabilitation, University of Florida, Jacksonville, FL, United States; 3 Physical Therapy, UF Health, University of Florida, Jacksonville, FL, United States; 4 Office of Research Affairs, College of Medicine, University of Florida, Jacksonville, FL, United States; 5 Department of Anesthesiology, University of Florida, Jacksonville, FL, United States

**Keywords:** inspiratory muscle training, diaphragm, mechanical ventilation, rehabilitation, total joint arthroplasty, respiratory strength, lung function

## Abstract

**Background:**

Total joint arthroplasty (TJA) is among the most common elective surgeries performed in the United States, and recent advances in perioperative care have shortened hospital stays and streamlined recovery. Yet, even brief periods of mechanical ventilation and anesthesia measurably weaken respiratory muscle function in the early postoperative period. Preoperative inspiratory strength training (IST) has been shown to reduce postoperative pulmonary complications in patients undergoing major cardiothoracic surgery, but whether these benefits extend to shorter procedures such as TJA, where hospital stays are already brief, remains unknown.

**Objective:**

This study aims to investigate whether preoperative IST prior to TJA surgery alters respiratory strength, lung function, and functional mobility. We hypothesize that preoperative IST is feasible and effective in increasing inspiratory strength and promoting the return of early postoperative respiratory function and functional mobility before hospital discharge.

**Methods:**

The study hypothesis will be tested using a randomized, prospective design at a single urban academic health system. Adults scheduled for TJA surgery will be randomized to either undergo daily IST (dIST), complete an acute IST (aIST) session in the preoperative area, or receive clinical standard of care (SOC). Inspiratory muscle strength and pulmonary function will be evaluated upon enrollment at a baseline assessment approximately 4 weeks before surgery and will be repeated on the day of surgery. Postoperative changes will be evaluated through the first 24 hours across 3 aims: to evaluate the feasibility and effectiveness of dIST for increasing preoperative respiratory strength (aim 1), to compare the effects of dIST and aIST on postoperative respiratory strength measures and lung function (aim 2), and to evaluate 24-hour functional outcomes (aim 3).

**Results:**

The study was funded in September 2023 and received institutional review board and safety officer approvals in December 2023. Enrollment began in March 2024, and as of May 2026, a total of 31 participants have been enrolled. Data analysis and publication of primary results are expected in 2027.

**Conclusions:**

This trial will provide the first controlled investigation of preoperative IST in TJA, offering early evidence on whether a low-cost, nonpharmacological intervention can preserve respiratory strength and accelerate functional recovery in this high-volume surgical population.

## Introduction

Total joint arthroplasty (TJA) surgeries are common elective surgeries that result in more than 1.3 million hospital stays in the United States annually [1], and surgical volume is expected to continue to grow through 2040 [2]. In the years since TJA surgeries transitioned away from being inpatient-only procedures, the hospital length of stay for TJA has progressively declined, and the prevalence of outpatient procedures has expanded [3]. In conjunction, clinical readiness for TJA has evolved to include optimizing preoperative education, implementing multimodal anesthesia and pain management, and initiating early lower extremity strengthening and walking rehabilitation [4-7]. However, an underappreciated aspect of TJA readiness and recovery is the potential impact of surgery on respiratory muscle capacity.

The primary inspiratory muscle, the diaphragm, undergoes acute remodeling in response to changes in activity (reviewed by Fogarty et al [8]). Hospital clinical practices such as mechanical ventilation, sedation, and anesthesia significantly decrease diaphragm recruitment, which can degrade ventilation and important related behaviors such as coughing [9]. Importantly, even short periods of mechanical ventilation typical of TJA surgeries contribute to impaired inspiratory force generation [10]. Lung volumes fall after TJA surgery and remain depressed 24 hours later [11]. The extent of the decline is most closely associated with older age [12], and acutely decreased lung function subsequently increases the risk for pulmonary complications [13].

Although postoperative pulmonary complications (PPCs) are relatively uncommon after TJA [14], they prolong the hospital stay, increase costs, and increase the mortality risk [14]. Older adults and those with preexisting lung disease and medical comorbidities may be more susceptible to respiratory weakness, poor cough function, and impaired oxygenation, which can interfere with postoperative rehabilitation and extend the projected length of stay [15,16]. In addition to patients with multiple preexisting health issues, people from racial and ethnic minority groups or those with low socioeconomic status also experience higher risks for acute complications after TJA [17-19]. Therapies designed to limit postoperative respiratory decline have the potential to address unmet needs and improve TJA outcomes for these underserved individuals.

Controlled studies in other surgical populations reveal significant clinical benefits of preoperative inspiratory strength training (IST) compared to usual surgical care. IST for 2-4 weeks before surgery significantly strengthens the respiratory muscles [20-24], mitigates surgical declines in lung function, and lowers the incidence of PPCs [22-26]. Additionally, strength gains associated with preoperative IST are associated with shorter lengths of stay [22,23], which may potentially reduce costs [23]. Although preoperative IST for cardiothoracic surgery is not in itself novel, its impacts on orthopedic surgeries are scarcely studied. We postulate that preoperative IST may be a powerful tool to preserve respiratory strength and cough force in people undergoing TJA. Aim 1 of this study is to investigate the effect of daily IST (dIST), delivered at high intensity preoperatively, on increasing respiratory strength and lung function before TJA.

Although preventative care and educational approaches are 2 recommended strategies to improve health [27], people from underserved communities may lack access to the resources necessary to attend appointments or struggle to complete unsupervised training. Lack of transportation, social support, and health literacy can impede regular adherence to a preoperative exercise plan [28]. As single intense inspiratory loading bouts transiently increase inspiratory pressure generation [29] and enhance central excitability [30], we postulate that they have the potential to counter the downregulation of respiratory neural drive during brief periods of mechanical ventilation. Thus, aim 2 of this study will evaluate whether short-term potentiation of inspiratory drive from a single acute IST (aIST) session immediately prior to surgery can attenuate postoperative declines in inspiratory pressure generation. As high-intensity IST programs that increase maximal inspiratory pressure (MIP) can enhance diaphragm fatigue resistance and reduce exercise-induced dyspnea [31,32], aim 3 of this study will evaluate whether either IST approach can improve functional mobility and dyspnea during the 24-hour postoperative rehabilitation session.

## Methods

### Trial Design

**Figure 1.**
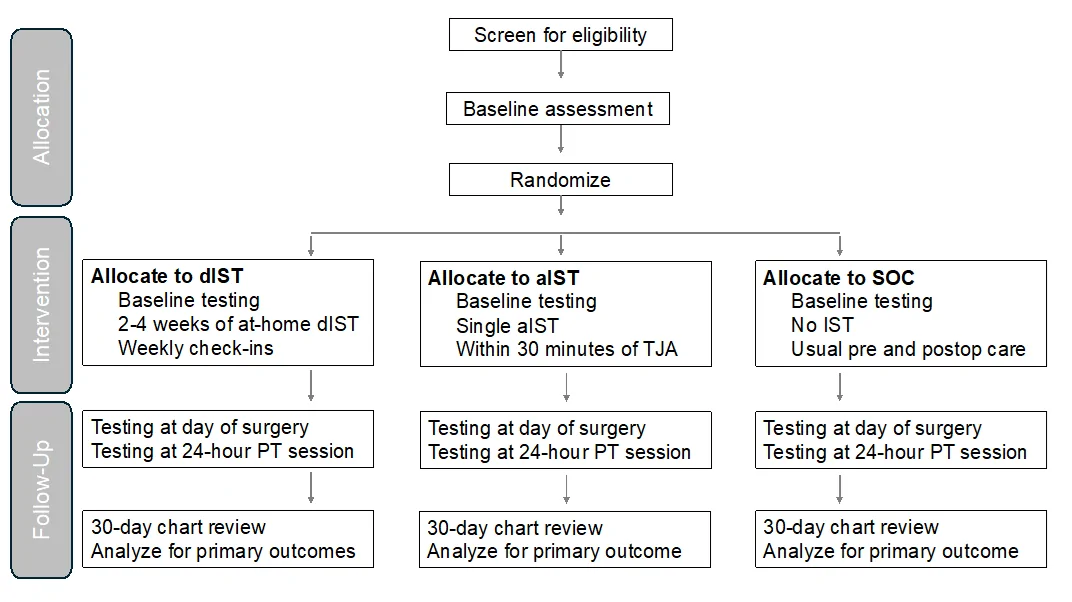
Projected flowchart of the clinical trial. aIST: acute inspiratory strength training; dIST: daily inspiratory strength training; IST: inspiratory strength training; PT: physical therapy; SOC: standard of care; TJA: total joint arthroplasty.

This is a randomized, prospective, single-blind clinical study. Eligible participants will be randomized to one of three study groups: (1) dIST, consisting of IST 5 days per week for a period of 2 to 4 weeks before surgery; (2) aIST, consisting of a single aIST session delivered just prior to surgery; or (3) standard of care (SOC), consisting of usual preoperative, surgical, and postoperative care. Participants will be tested at established time points beginning with a baseline preoperative visit approximately 1 month before surgery and will participate in tests through 24 hours following surgery. PPCs will be monitored for 30 days. This study is registered on ClinicalTrials.gov (NCT5381818), and trial reporting will be guided by the SPIRIT (Standard Protocol Items: Recommendations for Interventional Trials; Checklist 1) and CONSORT (Consolidated Standards of Reporting Trials; Checklist 2) procedures (Figure 1) [33]. Proof-of-concept data were collected with support from an internal pilot grant award, and the controlled study is being conducted with funding from the National Institute on Aging (NIA; R21 AG083667).

**Figure 2.**
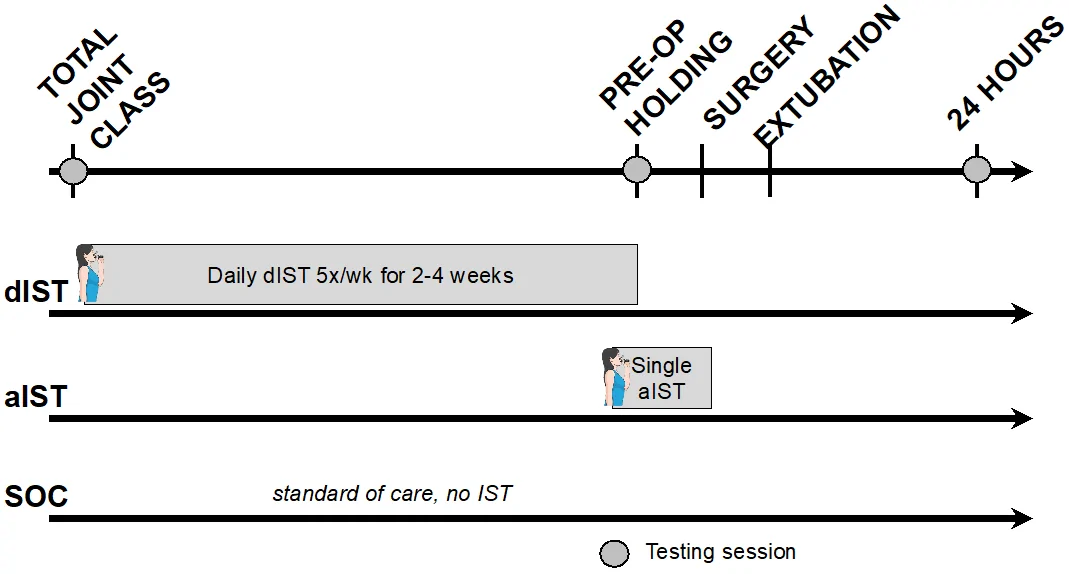
Outline of testing sessions and intervention groups. Participants will be randomized to 1 of 3 groups: daily inspiratory strength training (dIST) for 2-4 weeks before total joint arthroplasty, a single acute inspiratory strength training (aIST) session in the preoperative holding area, or standard of care (SOC; no training). Testing will occur upon enrollment, in the preoperative area, and 24 hours postoperatively. IST: inspiratory strength training.

The study will take place at a single academic health care system in the urban United States (Figure 2). Screening procedures will occur in the outpatient clinic, and preoperative and postoperative procedures will be conducted in the hospital setting. MIP, forced vital capacity (FVC), and the 6-minute walk distance (6MWD) test will be assessed at baseline during an initial outpatient assessment visit prior to total joint replacement (typically approximately 4-6 weeks before surgery and, when possible, timed to coincide with the preoperative “total joint education” clinical session). MIP and FVC will then be repeated on the day of surgery and 24 hours after extubation. The research team will track pulmonary complications based on the European Perioperative Clinical Outcomes (EPCO) definitions. Additionally, we will record clinical physical therapy (PT) documentation of participant vital signs, dyspnea, functional status (Activity Measure for Post-Acute Care [AM-PAC] “6-Clicks” mobility score, standard clinical care), and dyspnea and walking distance at the 24-hour PT session. Diaphragm ultrasound is an exploratory outcome measure that will be assessed in preoperative holding and 24 hours postoperatively. Participant confidentiality will be protected by assigning a unique study number to each participant and using the participant number for data collection. Data will be entered and stored in a REDCap (Vanderbilt University) database on a secure institutional drive.

### Participant Recruitment and Screening

Individuals with physician approval for elective joint replacement surgery who are interested in research participation will be referred to the study team by the Department of Orthopedics. We will enroll up to 60 participants. This includes 30% (18/60) participants who participated in nonrandomized, proof-of-principle pilot work for the purpose of estimating the sample size for the randomized study. Up to 70% (42/60) additional participants will be randomized, with the goal of completing procedures in 60% (36/60) randomized participants. The inclusion criteria are outlined in Textbox 1. Patients who meet the eligibility criteria and agree to participate in the study will sign a University of Florida Institutional Review Board (IRB)–approved informed consent form. The study coordinators will screen patients according to the inclusion and exclusion criteria via participant interview and medical record review.

Textbox 1. **Inclusion and exclusion criteria for study participation.**

**Inclusion criteria:**

- Aged 55 to 85 years at enrollment
- Scheduled for lower extremity joint arthroplasty surgery with a projected surgical time of >1 hour
- Able to follow instructions to complete inspiratory exercises
- Able to communicate adverse effects or a need for assistance
- One or more of the following risk factors for postoperative pulmonary complications:
  - Previous or current smoking
  - Diagnosis of pulmonary disease
  - Dyspnea with minimal exertion (ascending 1 flight of stairs)
  - Modified Frailty Index-5 score of ≥2
  - Impaired lung function (forced expiratory volume in 1 second and/or forced vital capacity <80% predicted)
  - Respiratory muscle strength below the lower limit of normal for age and sex

**Exclusion criteria:**

- American Society of Anesthesiologists physical status classification of ≥4
- Acute use of corticosteroids or antibiotics within the past 2 weeks
- Preoperative dependence on continuous supplemental oxygen
- Preoperative dependence on positive pressure breathing support while awake and upright (nighttime continuous positive airway pressure permitted)
- Progressive neuromuscular disease
- Participation in a pulmonary rehabilitation program
- Global Initiative for Chronic Obstructive Lung Disease classification ≥3, indicating severe obstructive pulmonary disease
- Active infectious disease requiring isolation (eg, COVID-19)

### Randomization and Blinding

Consenting patients who meet the inclusion criteria will be randomized to a study group according to a randomization sequence created using SAS (version 9.4; SAS Institute Inc). Due to the nature of the intervention, participants and the coinvestigator assigned to conducting the intervention will be aware of the group assignment. Surgeons and study evaluators will remain blinded to group assignment.

### Inspiratory Training Interventions

Participants will be randomized to the dIST, aIST, or SOC group.

#### dIST

The dIST group will complete preoperative IST for approximately 2 to 4 weeks before surgery. Participants will be given a portable device (EMST75/IA150, Aspire Products LLC) containing an adjustable-tension spring to provide a pressure-threshold training load. Participants will complete 5 sets of 5 maximal inspiratory breaths at maximal volume and speed, resting for approximately 1 minute between sets. A nasal clip will reduce pressure loss through the nose. The dIST training intensity will be 70% of the participant’s MIP, and the intensity will not be progressed further. The coinvestigator conducting the training will teach participants to use the IST device, complete the exercises, and record their work on a training log. The coinvestigator will call the patient at the first home IST session and weekly thereafter to address any questions that arise.

#### aIST

The aIST group will complete a single session of preoperative IST within 30 minutes prior to anesthesia induction, using a pressure-threshold training device (EMST75/IA150). Participants will complete 5 sets of 5 maximal inspiratory breaths at maximal volume and speed, with approximately 1 minute between sets. A nasal clip will be used to reduce pressure loss through the nose. The aIST target training intensity will be 70% of the participant’s MIP.

#### SOC

The SOC group will serve as the control group and will receive usual preoperative, surgical, and postoperative care. This includes the preoperative TJA class sponsored by the Department of Orthopedics, preoperative lower extremity strengthening home exercises, provision of an assistive device, and counseling for smokers to quit smoking preoperatively.

### Outcomes

#### Respiratory and Functional Assessments

A study evaluator blinded to group assignment will be responsible for conducting the assessments. All intervention groups will complete study assessments.

##### MIP

MIP is the primary outcome measure and represents the most negative pressure achieved by a participant during a maximal static inspiratory effort. Participants will be tested in accordance with American Thoracic Society (ATS) guidelines. MIP will be tested at the baseline assessment (approximately 4 weeks before surgery), in preoperative holding, and 24 hours postoperatively. Trials will be administered until a 10% variability is achieved across 3 trials (usually within 5 trials).

##### Spirometry

Spirometry is a secondary outcome measure. Test maneuvers will follow ATS guidelines [34] and shall include (1) FVC, the volume of air a person can forcefully and quickly exhale after taking a maximal inhalation; (2) forced expiratory volume in 1 second (FEV1), the volume of air forcefully exhaled during the first second of an FVC maneuver; (3) inspiratory capacity (IC), the volume of air inspired from end-expiration to total lung capacity; and (4) peak cough flow (PCF), the maximum airflow generated during a voluntary cough. Participants will be tested at the baseline assessment, in preoperative holding, and 24 hours postoperatively.

##### 6MWD

6MWD is a secondary outcome measure and represents the maximal distance walked by the patient during the 24-hour postoperative PT session, using the assistive device determined by the physical therapist to be most compatible with the patient’s underlying physical status and postoperative condition (pain and weight-bearing restrictions). In addition to the distance walked, the patient-reported primary limitation (eg, joint pain, weakness, or dyspnea), peak dyspnea level using the modified Borg scale [35], and vital signs will be recorded.

##### AM-PAC “6-Clicks” Functional Mobility

The 6-Clicks is a secondary outcome measure and assesses the difficulty or level of assistance needed for bed mobility, sit-to-stand, supine-to-sit, seated transfers, walking, and ascending stairs; scores are predictive of discharge needs after total knee and hip arthroplasty [36]. The 6-Clicks score is part of standard clinical care and will be recorded from the electronic health record at the 24-hour PT session.

##### Diaphragm Ultrasonography

Ultrasound is an exploratory outcome measure that will be used to calculate diaphragm thickness and thickening fraction during quiet breathing and maximal inspiratory efforts. Ultrasound procedures will follow the methods outlined by Sarwal et al [37].

### Feasibility and Safety Assessments

The electronic health record will be queried to obtain relevant information on patient, surgical, and postoperative information pertaining to the acute hospitalization, including clinical PT documentation of participant demographics, vital signs, dyspnea, functional status (AM-PAC “6-Clicks” functional mobility score), and walking distance at the 24-hour PT session. Feasibility of dIST and aIST is an exploratory aspect that will be assessed using the percentage of projected sessions completed by the participant, the proportion of participants assigned to dIST who are able to complete >75% of prescribed training sessions, and the average change in MIP between the baseline and day-of-surgery assessment points. To monitor PPCs, the study team will record relevant vital signs, requirements for supplemental oxygen, discharge information, and any pulmonary complications or unanticipated health care interventions needed up to 30 days postoperatively.

### Study Oversight

Participants will report levels of pain and dyspnea and undergo assessment of their vital signs before, during, and after study testing. Additionally, participants will also be asked to document their dyspnea during IST sessions and report any unusual symptoms during training. Safety will be monitored through adverse event reporting to the IRB, as well as reporting to an external safety officer. Adverse events will be tracked using a log. Nonserious and serious expected events will be reported biannually to the safety officer and annually to the IRB. Any adverse events determined to be serious, unexpected, and possibly related to study participation will be reported within 5 days to the IRB and within 48 hours to the safety officer and funding source. In addition to safety oversight, the IRB and funding agency will review study progress toward milestones at least annually and may elect to conduct random audits.

### Data Analysis

#### Sample Size Projection

The primary outcome measure is MIP. To project the sample size needed to detect changes in respiratory strength following dIST vs SOC, postoperative changes in MIP were obtained from a controlled, blinded study of IST in cardiac surgery [38]. Relative to baseline, postoperative MIP decreased by an average of 28 (SD 16) in the usual care group but only by 10 (SD 17) in the IST group. These data, along with similar publications of preoperative IST, demonstrate a moderate to large potential effect size to detect changes in MIP [22,24,26]. With a total of 36 participants (12 per group), a mixed-effects test could detect a 3×3 group-time interaction effect for training (partial η²=0.06, effect size (f)=0.253, α=0.05, 1−β=0.83).

#### Statistical Analysis

To test whether dIST or even a single aIST session can accelerate postoperative respiratory and functional recovery, the primary analysis will compare between-group changes using a mixed-effects model to account for within-subject correlation in repeated measurements. Fixed main effects will include intervention and time; the participant will be included as the random effect. If baseline measures differ between groups, an analysis of covariance (ANCOVA) will be used. The primary analyses will be (1) change in MIP for dIST (vs other groups) from baseline to the preoperative assessment and (2) change in MIP from the preoperative assessment to the postoperative assessment for dIST and aIST (vs SOC). Holm adjustments will be applied for multiple comparisons.

Feasibility will be characterized by the percentage of completed dIST sessions, diary completion, and attrition rate. These will be reported as descriptive variables. The prevalence of PPCs will be compared between groups using chi-square or Fisher exact tests, as appropriate.

#### Data Management

The original dataset will be maintained at the investigator’s institution on a password-protected, encrypted drive and backed up to a secure server. Protocols and informed consent forms for participation in the study will be shared with the deidentified data through a National Institutes of Health (NIH)–approved data repository. Recruitment progress and final results will be posted for public access at ClinicalTrials.gov.

### Ethical Considerations

The study was approved by the IRB of the University of Florida (IRB202102681). The informed consent document was reviewed and approved by the University of Florida IRB. Informed consent will be obtained from all participants involved in the study.

## Results

The study was funded in September 2023. IRB approval was obtained in December 2023, and procedures were approved by the safety officer that month. The first randomized participant was enrolled in March 2024. As of May 2026, 31 participants have been enrolled. Data analysis and publication of the primary study results are expected in 2027.

## Discussion

Despite strong evidence demonstrating the benefits of IST on respiratory strength, mobility, and PPCs after major cardiothoracic surgeries [21-24], little is known about its potential to offset surgical declines and accelerate recovery following TJA. This study focuses on TJA because of its high volume and expedited discharge pathway, with hospital lengths of stay averaging <1.5 days for total knee and hip surgeries [39] and anticipated to further decrease in the future [3]. Despite efforts to maximize efficiency of these surgical interventions, certain people with preexisting lung disease, multiple comorbidities, and frailty will require preemptive monitoring due to increased risk for complications. Preoperative IST is an inexpensive, nonpharmacological intervention with the potential to enhance preoperative readiness. As we cannot precisely control the exact timing and number of preoperative dIST sessions, the statistical analysis will explore the volume of dIST as a potential covariate of the primary study end points.

The inclusion of a single, intensive IST session in preoperative holding is a novel aspect of this study. Intense respiratory loading exercises induce temporary increases in respiratory neural drive that could potentially counter the reported decreases in diaphragm contractile function during controlled mechanical ventilation [29,30]. Although single exercise bouts are not expected to achieve lasting gains in function, even a temporary countermeasure may be advantageous for higher-risk surgical candidates who lack the ability to complete weeks of preparatory exercise, whether due to a lack of transportation to attend appointments, challenges with health literacy, or the need for an unplanned or emergent surgery.

The primary outcome of this trial is MIP, a clinical estimate of strength. However, additional secondary measures were included to investigate the effects of the interventions on early postoperative activity-based measures such as coughing force, walking distance, and functional mobility, along with patient-reported dyspnea. Postoperative walking and functional mobility are important functional limitations following TJA that influence the timing of disposition and postacute rehabilitation needs [40]. Although the projected effect size of preoperative IST appears robust for impairment-level outcomes, this study will also provide early indication of its ability to carry over to functional activities and participation-level recovery. As preoperative 6MWD can predict functional ambulation after total joint surgeries [41], the statistical plan will explore preoperative walking distance as a covariate.

As a first preliminary study in TJA, some aspects of the study design remain limited. The sample size was projected from published work in cardiac surgery to detect changes in MIP between dIST and no preoperative exercise. Although we anticipate gaining valuable feasibility information regarding completion of aIST in the acute preoperative setting, we may lack the power to comprehensively distinguish which preoperative training approach (dIST or a single aIST session) can more effectively retain postoperative MIP and functional mobility. Postoperative walking and functional mobility at 24 hours can be influenced by a multitude of factors beyond respiratory strength and may include variations in pain management and pain perception, the ability to participate in same-day therapy, and lack of sleep, nutrition, or hydration. Blinded evaluators will plan for premedication with nursing before the planned 24-hour PT session and ensure that the timing does not interfere with meals. Participants randomized to SOC did not receive a respiratory trainer or training diary or undergo weekly check-ins, and the absence of a sham or attentional control group could inadvertently introduce expectation bias. Even in higher-risk patients, there may be few PPCs in the 30-day period following TJA. However, monitoring complications will enable us to further explore the prevalence of postoperative pulmonary issues in this cohort. Surgeons and study evaluators will be blinded to group assignment, but patients and other study staff will, by necessity, know the allocation.

Research dissemination will be directed toward health care specialties involved in acute TJA management. Study results will be communicated through presentations at scientific conferences and will be published in peer-reviewed journals directed toward readers in orthopedic surgery, rehabilitation, and anesthesiology.

To conclude, this investigator-initiated, randomized, single-blind trial is designed to evaluate the effects of preoperative IST on measures of respiratory strength and function, walking and mobility, and postoperative recovery from lower extremity TJA. Completion of the study will help illuminate whether dIST can improve MIP and cough efficacy preoperatively, whether daily or single-session inspiratory strengthening can attenuate postoperative declines in respiratory strength and pulmonary function, and whether group differences in postoperative inspiratory strength promote earlier recovery of functional mobility.

## Supplementary material

10.2196/103290 Checklist 1 SPIRIT checklist.

10.2196/103290 Checklist 2 CONSORT 2025 checklist.

10.2196/103290 Peer Review Report 1 Peer-review report from MRS - Musculoskeletal Rehabilitation Sciences Study Section, Musculoskeletal, Oral and Skin Sciences Integrated Review Group, National Institute on Aging (National Institutes of Health, USA).

## Abbreviations

6MWD: 6-minute walk distance
aIST: acute inspiratory strength training
AM-PAC: Activity Measure for Post-Acute Care
ANCOVA: analysis of covariance
ATS: American Thoracic Society
CONSORT: Consolidated Standards of Reporting Trials
dIST: daily inspiratory strength training
EPCO: European Perioperative Clinical Outcomes
FEV1: forced expiratory volume in 1 second
FVC: forced vital capacity
IC: inspiratory capacity
IRB: Institutional Review Board
IST: inspiratory strength training
MIP: maximal inspiratory pressure
NIA: National Institute on Aging
NIH: National Institutes of Health
PCF: peak cough flow
PPC: postoperative pulmonary complication
PT: physical therapy
SOC: standard of care
SPIRIT: Standard Protocol Items: Recommendations for Interventional Trials
TJA: total joint arthroplasty
