# Supplementary material for: Inspiratory Muscle Training Before Total Joint Arthroplasty to Improve Postoperative Outcomes: Protocol for a Randomized Controlled Trial
Source: JMIR Res Protoc. 2026 Sep 11;15:e103290. doi: 10.2196/103290 (PMC13566066; doi:10.2196/103290)
Supplement: Supplementary file 2 [file resprot-v15-e103290-s003.pdf]

SUMMARY STATEMENT
(Privileged Communication)

PROGRAM CONTACT:
LYNDON JOSEPH
301-496-6926
josephlj@mail.nih.gov

Release Date: 03/14/2023
Revised Date:

Application Number: 1 R21 AG083667-01
Formerly: 1R21AR083037-01

Principal Investigator

SMITH, BARBARA K

Applicant Organization: UNIVERSITY OF FLORIDA

Review Group: MRS
Musculoskeletal Rehabilitation Sciences Study Section

Meeting Date: 02/16/2023
Council: MAY 2023
Requested Start: 07/01/2023
RFA/PA: PA20-194
PCC: 4AGERLJ
Dual IC(s): HD

Project Title: Inspiratory Muscle Training to Improve Total Joint Arthroplasty Outcomes

SRG Action: Impact Score:29 Percentile:12 +
Next Steps: Visit https://grants.nih.gov/grants/next_steps.htm
Human Subjects: 30-Human subjects involved - Certified, no SRG concerns
Animal Subjects: 10-No live vertebrate animals involved for competing appl.
Gender: 1A-Both genders, scientifically acceptable
Minority: 1A-Minorities and non-minorities, scientifically acceptable
Age: 3A-No children included, scientifically acceptable

Project Year	Direct Costs Requested	Estimated Total Cost
1	150,000	227,032
2	125,000	189,193
TOTAL	275,000	416,225

ADMINISTRATIVE BUDGET NOTE: The budget shown is the requested budget and has not been adjusted to reflect any recommendations made by reviewers. If an award is planned, the costs will be calculated by Institute grants management staff based on the recommendations outlined below in the COMMITTEE BUDGET RECOMMENDATIONS section.

1R21AG083667-01 Smith, Barbara

RESUME AND SUMMARY OF DISCUSSION: The effects of total joint arthroplasty (TJA) surgery on pulmonary and respiratory muscle function are not well appreciated, but aspects such as mechanical ventilation, anesthesia, and opioid medications can negatively affect respiration and put one at risk for pulmonary complications. This is a new clinical trial R21 application that proposes to evaluate whether targeted respiratory muscle exercises preserve breathing function, reduce shortness of breath, and accelerate mobility after TJA surgery. Reviewers expressed enthusiasm for the significance of the problem and rigor of prior research; outcomes related to improvements after cardiac surgery were compelling. Additional strengths include a strong team qualified to do the work, a supportive environment with the necessary resources, and the focus on an underserved and vulnerable population. The translation of this work from cardio-thoracic surgery to a new population is innovative, and reviewers noted appropriate outcome measures, good power analysis, a good experimental design with blinding, and solid preliminary data that support the hypothesis. Feasibility could be an issue with this population, but the proposal addresses issues related to access, using home telehealth and a single session only if needed. Weaknesses were also discussed, such very broad inclusion criteria for such a small group, making comparisons difficult (could be varying amounts of time under anesthesia based on which joint, different comorbidities, etc.). Some reviewers were concerned that the success from previous studies in cardiac surgery might not convey to lower extremity orthopedic surgeries, but others noted data supports the likelihood of success. All the reviewers agreed that aim 3 is the weakest aim, but likely could be sorted out by this team. Following discussion, the panel agreed that the strengths in the significance, investigative team, and innovation outweighed the minor weaknesses noted in the approach. There was strong enthusiasm for this application and the overall impact was assessed as high.

DESCRIPTION (provided by applicant): Total joint arthroplasty (TJA) surgeries are among the most common elective surgeries in the US and projected to increase in frequency. Recent TJA clinical practices have effectively reduced post-operative hospital length of stay, yet despite improvements in the efficiency of TJA care, the effects of TJA surgical procedures on pulmonary and respiratory muscle function are less widely appreciated. Even during surgeries as short as TJA, the administration of anesthesia, neuromuscular inactivity, and mechanical ventilation acutely deteriorate pulmonary function and trigger rapid, significant proteolysis of the primary inspiratory muscle, the diaphragm. However, conventional TJA preoperative care does not conventionally address these issues. Older adults, smokers, and those with significant pre-existing lung disease, multiple medical comorbidities, or socioeconomic disadvantage face the greatest risk for declines in post-operative pulmonary and respiratory neuromuscular function. Most patients who utilize our urban safety-net academic medical center have at least one of these risk factors, which can interfere with acute rehabilitation, increase the risk for post-operative pulmonary complications, and extend hospital length of stay. Thus, we propose a clinical study of preoperative inspiratory muscle training (IMT) among individuals with increased risk for pulmonary post-operative complications. Preoperative IMT has been shown to counteract post-operative inspiratory weakness and reduce postoperative pulmonary complications following prolonged cardiac surgeries, but its potential benefits have not been investigated in shorter surgeries such as TJA, with expected brief post-operative hospitalizations. The central hypothesis of this project is that preoperative IMT is feasible and will improve respiratory strength, hasten the early postoperative respiratory recovery, and optimize functional mobility for hospital discharge. Adults scheduled for TJA with pre-existing respiratory muscle or lung impairment will be randomized to complete either: daily IMT in advance of surgery (dIMT), a single acute IMT session immediately before surgery (aIMT), or usual surgical standard of care (SOC). Follow-up testing on the day of surgery and during the acute post-operative hospitalization will identify the feasibility of IMT (Aim 1), distinguish IMT effects on inspiratory

and cough strength, (Aim 2), and evaluate patient readiness for discharge (Aim 3). This high risk, proof-of-principle proposal will provide the first controlled evidence concerning disturbances in the regulatory functions of breathing following TJA. Our plan is that data generated from this study will form the basis for future mechanistic studies of IMT to restore breathing strength and further optimize early rehabilitation following TJA.

PUBLIC HEALTH RELEVANCE: The proposed research will evaluate whether targeted respiratory muscle exercises preserve breathing function, reduce shortness of breath, and accelerate mobility after total joint replacement surgery. The proposal is relevant to public health because the knowledge learned it will increase our understanding of the impacts of surgery on breathing and could lead to improved approaches to prepare and recover from total joint replacement surgery.

CRITIQUE 1

Significance: 1
Investigator(s): 2
Innovation: 1
Approach: 3
Environment: 1

Overall Impact: This is a new R21 clinical trial from an established investigator. It is a prospective, controlled clinical trial designed to test if the proposed intervention, preoperative inspiratory muscle training, can reduce post-operative complications in vulnerable patients following total joint arthroplasty. Aim 1 will first test whether a pre-operative regimen of 2-4 weeks of daily inspiratory muscle training before a scheduled TJA improves pulmonary function in a population of patients with one or more risk factors for postoperative pulmonary complications, including whether this intensive of a treatment dose is feasible for patients who typically receive care at an urban safety-net medical center. There will be one baseline visit with training, and then remote instruction and guidance. Because of the expectation that many people may not be able to complete the full regimen, Aim 2 tests whether a single treatment (termed acute IMT), provided on the day of surgery, will improve lung function. In the same population of participants, Aim 3 will test whether any respiratory benefits observed in Aims 1 and 2 translate into improved “functional readiness” for discharge after surgery.

The scientific rigor of the approach is high. Assessors of outcome metrics and the PI are blinded to group. 36 subjects will be randomized to 3 groups – the daily group, the acute group, and a control group that receives standard of care. The experimental design was powered for a medium effect size based on the results of controlled studies of the effects of the interventions of cardiothoracic surgery. This project is proposed as a high-risk/high-reward pilot study to provide initial data from which to design future-controlled studies to optimize results.

This proposal effectively argues this is a significant problem, and a major score driver is the strong scientific rigor of prior work. The same types of interventions have been shown in controlled scientific studies to improve lung function and lower the incidence of post-op pulmonary complications in cardiothoracic surgeries. Despite being presented as a pilot study, there is pilot data that generally supports the hypotheses. The application of these methods to TJA, the focus on an underserved population, and the attempts to define attainable interventions are innovative.

The investigative team is strong and uniquely suited to the completion of this work.

1. Significance:

Strengths

- Significance of the problem is high: mortality after TJA, an overwhelmingly successful surgery when there are no post-op complications, is 250x higher if there are post-op complications. Post-op complications are common.
- Prior research in support of the application includes multiple controlled studies that demonstrate the proposed intervention strengthens respiratory muscles, mitigates declines in lung function, and lowers the incidence of post-op pulmonary complications in cardiothoracic surgeries.
- Critical barrier to progress in the field seems to be the need to demonstrate the same pre-operative therapies used to avoid post-op lung complications in cardiovascular will have similar effects in orthopaedic surgeries. There are few studies, and while what is published lends support, there are gaps in the literature.

Weaknesses

- None noted.

2. Investigator(s):

Strengths

- PI and co-I well suited to the project

Weaknesses

- [Minor] Explicit mention of statistical analysis by a colleague without letter of support or biosketch included. University of Florida has a statistical consulting center (BERD), which indicates they have access to statistical support and services for a fee, so concern is small.

3. Innovation:

Strengths

- Innovation is to adopt a methodology that has been demonstrated to reduce post-op complications following cardiothoracic surgery for common orthopaedic surgery, total joint replacement.
- Innovation is focus on a population that has high need.
- Innovation includes investigating single impact of single IMT session, given the socioeconomic/access to medical care challenges of the target population.

Weaknesses

- None noted.

4. Approach:

Strengths

- Assessors and PI are blinded to group.
- Control group.
- Powered for a medium effect size, based on observed difference in cardiac surgery.
- Pilot data demonstrating dimt and aimt maintained MIP while control group dropped 10%.

- Looking at aIMT, with the eye toward not everyone will be able to/complete dIMT – rationale for benefit is the neural effect, supported by literature.
- SABV: aiming for 50/50 split between genders, propose secondary analysis to evaluate effects – appropriate for R21 mechanism, state current work.

Weaknesses

- 6-minute walk test post-op – confound with orthopaedic outcome?
- Not clear how many subjects/who they were in the pilot data for Aim 1.
- Pilot data for Aim 2 (Fig 4) is not significant.
- Aim 3 likely confounds with surgical outcome – some acknowledged (Pain). Minor weakness, given the R21 mechanism and likelihood data collected in Aim 3 will be informative for future studies.

5. Environment:

Strengths

- Strong environment

Weaknesses

- None noted.

Study Timeline:

Strengths

- Timeline is appropriate for the proposed work

Weaknesses

- None noted.

Protections for Human Subjects

Acceptable Risks and/or Adequate Protections

Data and Safety Monitoring Plan (Applicable for Clinical Trials Only):

Acceptable

Inclusion Plans:

- Sex/Gender: Distribution justified scientifically
- Race/Ethnicity: Distribution justified scientifically
- For NIH-Defined Phase III trials, Plans for valid design and analysis: Not applicable
- Inclusion/Exclusion Based on Age: Distribution justified scientifically
- Strength of the proposal is its focus on vulnerable population, focus on underserved population who access medical care via an urban-safety net hospital

Vertebrate Animals

Not Applicable (No Vertebrate Animals)

Biohazards

Not Applicable (No Biohazards)

Applications from Foreign Organizations

Not Applicable (No Foreign Organizations)

Select Agents

Not Applicable (No Select Agents)

Resource Sharing Plans

Acceptable

Authentication of Key Biological and/or Chemical Resources

Not Applicable (No Relevant Resources)

Budget and Period of Support

Recommend as Requested

CRITIQUE 2

Significance: 3
Investigator(s): 2
Innovation: 4
Approach: 5
Environment: 1

Overall Impact: This proposal addresses the often-overlooked issue of pulmonary complications after surgical procedures requiring significant time under anesthesia with mechanical ventilation. Specifically, the investigators seek to evaluate the effects of pre-operative inspiratory muscle training on pulmonary and respiratory function after total joint arthroplasty. The project is not technically innovative, as these approaches have been demonstrated to be effective for reducing pulmonary complications after cardiac surgeries; however, this is a minor concern as this will be the first controlled study of respiratory training for orthopedic surgeries. The proposal also effectively addresses several important barriers to access and inclusion to therapy through providing telehealth solutions and evaluating the impact of single versus repeated exposures to therapy. The score-driving concerns are the broad inclusion criteria (*i.e.,* total hip and knee replacements) and whether the sample size and choice of outcome measures will be sufficient to address the proposed aims. Overall enthusiasm is still moderately high given the importance of the problem and the compelling design that will facilitate clinical translation.

1. Significance:

Strengths

- Pulmonary complications are relatively frequent after total joint replacement surgeries, with significant negative effects of mortality, cost, and time in hospital. Further, preliminary evidence suggests these effects may be amplified in underserved populations.
- Preliminary evidence suggests that respiratory function is impaired for over 20% of individuals at 24 hours post-surgery and that inspiratory muscle training is well received and may support post-operative function.

Weaknesses

- The scientific rigor of evidence for whether or how outcomes may differ for lower-extremity total joint arthroplasty versus cardiothoracic and scoliosis surgeries is not well defined. Whether the proximity of the surgery to respiratory systems impacts or amplifies expected effects of anesthesia on respiratory function or post-operative effects of muscle training were unclear.

2. Investigator(s):

Strengths

- The PI has demonstrated expertise in diaphragm function and respiratory therapy which will support the execution and clinical translation of the proposed work. She has assembled a strong team, including a co-investigator in anesthesiology with extensive complementary expertise, as well as the research coordinators, therapists, and orthopedic team to support recruitment and execution.

Weaknesses

- No clear statistical support beyond evaluation of study design by the university Biostatistics & Epidemiology Research Core. Their ongoing involvement and support of the complex methods to analyze this data and support future studies are unclear.

3. Innovation:

Strengths

- The proposed work would represent the first controlled study of inspiratory muscle training for orthopedic surgery and test the broader effects of anesthesia and pulmonary therapy on post-operative function.

Weaknesses

- Although the project deploys a therapeutic approach to a new population, it is questionable if this work represents a high-risk, high-reward endeavor.

4. Approach:

Strengths

- The focus on developing a protocol that will support translation and access to diverse populations is a strength. By testing the feasibility of telehealth support of inspiratory muscle training and single-session effects, the impacts of the proposed work are amplified.
- The overall study design is clear and includes a broad suite of outcomes to address different aspects of therapy deployment and respiratory function.

Weaknesses

- The broad inclusion criteria (e.g., including both hip and knee procedures, and presumably, unilateral and bilateral procedures) is a concern as it introduces significant variability in the small, proposed study population that will likely make it challenging to evaluate the effects of inspiratory muscle training on post-operative function.
- While some pilot work has been conducted and appears to show promise, it was challenging to understand and interpret in context of the proposed work. The number of participants tested in the pilot for Aim-1 (*e.g.,* Fig. 3) and who was included in this population was unclear. Similarly, for Aim-3, it is challenging to compare the standard of care and therapy groups with only two subjects and no details on the surgery types, which have significant impacts on walking function.
- The choice of 6-min walk test for Aim-3 goals was not well justified and will be highly dependent on the type/scope of surgery. Other values of cardiopulmonary function during walking may be more related to the goals of the proposal.
- The sample size is small (N=12) for each group and power analysis seems to be based on a limited set of the proposed aims and a different study population. Whether the existing pilot data was also considered to inform these calculations or how accounting for other factors (e.g., surgery type, sex, age) would influence projected power to address the aims was not well addressed.
- Whether or how follow-up for pulmonary complications beyond the 24-hour evaluation would be collected or evaluated was not clear but seems important to inform future studies.

5. Environment:

Strengths

- Outstanding environment at the University of Florida, especially with the BREATHE Center which is devoted to research on pulmonary function. All of the required equipment, resources, and recruitment mechanisms appear available to support the proposed work.

Weaknesses

- None noted.

Study Timeline:

Strengths

- Realistic recruitment based on clinic number and past surgical intakes.

Weaknesses

- Short timeline for data analysis and dissemination.

Protections for Human Subjects

Acceptable Risks and/or Adequate Protections

- Adequate consideration of risks and protocol design.

Data and Safety Monitoring Plan (Applicable for Clinical Trials Only):

Acceptable

- Limited description of data availability, but adequate for small study design.

Inclusion Plans

- Sex/Gender: Distribution justified scientifically
- Race/Ethnicity: Distribution justified scientifically
- For NIH-Defined Phase III trials, Plans for valid design and analysis:
- Inclusion/Exclusion Based on Age: Distribution justified scientifically
- Adequately addresses sex as a biology variable as well as inclusion based on race, ethnicity, and age.

Vertebrate Animals

Not Applicable (No Vertebrate Animals)

Biohazards

Not Applicable (No Biohazards)

Applications from Foreign Organizations

Not Applicable (No Foreign Organizations)

Select Agents

Not Applicable (No Select Agents)

Resource Sharing Plans

Acceptable

Authentication of Key Biological and/or Chemical Resources

Acceptable

Budget and Period of Support

Recommend as Requested

CRITIQUE 3

Significance: 3
Investigator(s): 3
Innovation: 2
Approach: 1
Environment: 1

Overall Impact: This R21 proposes a clinical study of preoperative inspiratory muscle training for those with risk factors for postoperative complications. Total joint arthroplasties are common and while improved clinical approaches have reduced post-operative hospital stays, the effects on pulmonary function due to anesthesia and mechanical ventilation affect breathing and the diaphragm are marked but under-considered in clinical care. The proposed use of IMT prior to surgery is a new application for short surgeries but has been previously applied with success for longer cardiac surgeries. There is also an innovative trial of acute IMT, in contrast to more typical daily IMT, that has high potential to have more participant compliance especially among underserved populations with difficulty attending the clinic for a longer-term regime. There is a strong team of researchers in an excellent environment with a very clearer designed and motivated study protocol. Preliminary data suggest a high likelihood of both respiratory and functional impacts from even the acute regime. Minor weaknesses (e.g., no formal co-investigator from orthopaedics) do not significantly dampen enthusiasm for this high impact proposal.

1. Significance:

Strengths

- Respiratory impairments occur even during relatively short TJA procedures and persist afterwards and increase the risk for pulmonary complications during an extremely common class of surgeries. IMT is used prior to cardiac surgery with promising effects on airway clearance and reduced complications, which has been shown in multiple controlled studies. The use of IMT has not been previously examined in TJA, which is on a shorter timescale. Standard TJA practices like incentive spirometry do not prevent respiratory weakness.
- There is clear literature motivation that the time scale of TJA is sufficient to impair respiratory function due to mechanical ventilation, opioids, and neuromuscular inactivity, and that forced vital capacity and expired volume are depressed following TJA.
- Pilot data reveal impaired respiratory muscle function reduced 20% at 24 hours after TJA, but IMT was tolerated and preserved pressure generation, which provides important evidence that IMT should be further explored, since a successful IMT regime could improve post-operative respiratory function and reduce hospital stays, morbidity, and costs.
- The risk for acute complications in the population served by the research institution specifically are higher, and therapies designed to improve postoperative respiratory function targets this group. The exploration of acute IMT that may be more practical than longer term training in an at-risk population with limited transportation; this is a strong translational strength.
- Limitations to prior work applying IMT to orthopaedic surgeries for scoliosis include not observing pulmonary complications and no control group. These will be addressed in the current study.

Weaknesses

- It is not clear whether there is any reason why IMT would not be physically tolerated pre-operatively in this group in some way that differs from cardiac patients who tolerate it well.

2. Investigator(s):

Strengths

- Dr. Smith brings expertise in diaphragm function, FES, and noninvasive assessments of diaphragm function appropriate to the proposed work. Dr. Baunecker is an anesthesiologist with identifying risks for post-operative respiratory dysfunction.
- Staff is planned for research coordinator roles and a research physical therapist.

- There are letters of support from two assistant professors in the department of orthopaedic surgery committing access to patients and participation in the study.

Weaknesses

- No consultant or co-investigator partner from orthopaedics is formally participating in this project studying the effects for TJA (minor).

3. Innovation:

Strengths

- This is a controlled study of preop IMT as applied to orthopaedic surgeries, which has not previously explored with IMT. The comparison of aIMT to daily IMT provides an important insight into the intensity of IMT needed to realize respiratory benefits. Exploratory subanalyses will examine factors related to feasibility including intensity, surgical factors, demographics, and biological variables that are critical to identify the scenarios and patients most likely to benefit.

Weaknesses

- None noted.

4. Approach:

Strengths

- The comparisons of daily IMT, acute IMT, or standard of care is an important controlled design that will allow for insight into the intensity of training needed for benefits. Outcomes measures are reasonable, including inspiratory and cough strength, patient readiness for discharge, and feasibility of IMT. More advanced functional analyses using diaphragmatic ultrasonography to examine muscle stiffness (related to pressure) and excursion are also included and provide exploratory evidence that will bolster future mechanistic studies. The planned protocol adds a control and randomized design not present in prior work on IMT applied to scoliosis research.
- 36 adults undergoing lower limb TJA with surgery durations >1 hour and who have risk factors for pulmonary complications are planned; the design is appropriately powered and inclusion and exclusion criteria are reasonable.
- The plans to address logistical feasibility are key to future translational impact, and are well thought out. For example, pilot work showed that 50% of eligible patients declined IMT because of travel; thus the proposed project budgets travel funds. Diaries and trainers will be used to record compliance/feasibility.
- Pilot work in aIMT provides strong evidence of the likely impact of the proposed work. Pilots with aIMT in a cardiac population showed quicker extubation, improved response to phrenic nerve stimulation, and preserved post op inspiratory force. Pilots with aIMT in a TJA population improved functional outcomes (e.g., 6 minute walk substantially).
- BME, age, race, sex, and baseline strength will be evaluated. Pain will be considered as a confounding variable.

Weaknesses

- None noted.

5. Environment:

Strengths

- The environment is appropriate for the planned work. In particular, the resources for Biostatistics, Epidemiology and Research Design, Center for Breathing Research and Therapeutics, and health informatics infrastructure supports the planned activities. The necessary equipment for spirometry, handgrip dynamometry, and other assessments are available.

Weaknesses

- None noted.

Study Timeline:

Strengths

- The study timeline is sufficiently detailed and includes regulatory approvals, training and start up milestones, as well as data collection and analysis/interpretation/dissemination milestones. The timeline is appropriate.

Weaknesses

- None noted.

Protections for Human Subjects

Acceptable Risks and/or Adequate Protections

- Protection plans are throrough and appropriate.

Data and Safety Monitoring Plan (Applicable for Clinical Trials Only):

Acceptable

- Plan is appropriate and regular meetings to review progress and safety are planned.

Inclusion Plans

- Sex/Gender: Distribution justified scientifically
- Race/Ethnicity: Distribution justified scientifically
- For NIH-Defined Phase III trials, Plans for valid design and analysis: Not applicable
- Inclusion/Exclusion Based on Age: Distribution justified scientifically
- Older adults are the focus due to the prevalence of TJA in this group. Women and minorities will be included.

Vertebrate Animals

Not Applicable (No Vertebrate Animals)

Biohazards

Not Applicable (No Biohazards)

Applications from Foreign Organizations

Not Applicable (No Foreign Organizations)

Select Agents
Not Applicable (No Select Agents)

Resource Sharing Plans
Acceptable

Authentication of Key Biological and/or Chemical Resources
Acceptable

Budget and Period of Support
Recommend as Requested

THE FOLLOWING SECTIONS WERE PREPARED BY THE SCIENTIFIC REVIEW OFFICER TO SUMMARIZE THE OUTCOME OF DISCUSSIONS OF THE REVIEW COMMITTEE, OR REVIEWERS' WRITTEN CRITIQUES, ON THE FOLLOWING ISSUES:

PROTECTION OF HUMAN SUBJECTS: ACCEPTABLE

INCLUSION OF WOMEN PLAN: ACCEPTABLE

INCLUSION OF MINORITIES PLAN: ACCEPTABLE

INCLUSION ACROSS THE LIFESPAN: ACCEPTABLE

COMMITTEE BUDGET RECOMMENDATIONS: The budget was recommended as requested.

Footnotes for 1 R21 AG083667-01; PI Name: Smith, Barbara K

+ Derived from the range of percentile values calculated for the study section that reviewed this application.

NIH has modified its policy regarding the receipt of resubmissions (amended applications).See Guide Notice NOT-OD-18-197 at https://grants.nih.gov/grants/guide/notice-files/NOT-OD-18-197.html. The impact/priority score is calculated after discussion of an application by averaging the overall scores (1-9) given by all voting reviewers on the committee and multiplying by 10. The criterion scores are submitted prior to the meeting by the individual reviewers assigned to an application, and are not discussed specifically at the review meeting or calculated into the overall impact score. Some applications also receive a percentile

ranking. For details on the review process, see
http://grants.nih.gov/grants/peer_review_process.htm#scoring.

MEETING ROSTER

Musculoskeletal Rehabilitation Sciences Study Section
Musculoskeletal, Oral and Skin Sciences Integrated Review Group
CENTER FOR SCIENTIFIC REVIEW
MRS
02/16/2023 - 02/17/2023

Notice of NIH Policy to All Applicants: Meeting rosters are provided for information purposes only. Applicant investigators and institutional officials must not communicate directly with study section members about an application before or after the review. Failure to observe this policy will create a serious breach of integrity in the peer review process, and may lead to actions outlined in NOT-OD-22-044 at https://grants.nih.gov/grants/guide/notice-files/NOT-OD-22-044.html, including removal of the application from immediate review.

CHAIRPERSON(S)

WARD, SAMUEL RICHARD, PHD
PROFESSOR AND VICE CHAIR
DEPARTMENT OF ORTHOPAEDIC SURGERY,
RADIOLOGY AND BIOENGINEERING
UNIVERSITY OF CALIFORNIA, SAN DIEGO
LA JOLLA, CA 92093

MEMBERS

ALBERTS, JAY L, PHD
THE EDWARD AND BARBARA BELL ENDOWED CHAIR FOR PARKINSON'S RESEARCH
STAFF, DEPARTMENT OF BIOMEDICAL ENGINEERING
CENTER FOR NEUROLOGICAL RESTORATION
CLEVELAND CLINIC
CLEVELAND, OH 44195

ANDERSON, ANDREW EDWARD, PHD
PROFESSOR
DEPARTMENT OF ORTHOPEDICS
UNIVERSITY OF UTAH
SALT LAKE CITY, UT 84108

ASHTON-MILLER, JAMES ANTHONY, PHD
PROFESSOR
DEPARTMENT OF MECHANICAL ENGINEERING
COLLEGE OF ENGINEERING
UNIVERSITY OF MICHIGAN
ANN ARBOR, MI 48109

BOYER, KATHERINE ALAINE, PHD
ASSOCIATE PROFESSOR
DEPARTMENT OF KINESIOLOGY
SCHOOL OF PUBLIC HEALTH AND HEALTH SCIENCE
UNIVERSITY OF MASSACHUSETTS-AMHERST
AMHERST, MA 01003

BOYNE, PIERCE, PHD *
ASSOCIATE PROFESSOR
DEPT OF REHABILITATION, EXERCISE AND NUTRITION SCIENCES, COLLEGE OF ALLIED HEALTH SCIENCES, INTERDISCIPLINARY NEURORECOVERY LABORATORY
UNIVERSITY OF CINCINNATI
CINCINNATI, OH 45221

DORSEY, KRISTEN LAUREN, PHD *
ASSOCIATE PROFESSOR
ELECTRICAL AND COMPUTER ENGINEERING
PHYSICAL THERAPY AND REHABILITATION SCIENCES
COLLEGE OF ENGINEERING
NORTHEASTERN UNIVERSITY
BOSTON, MA 02115

GAFFNEY, BRECCA, PHD *
ASSISTANT PROFESSOR
DEPARTMENT OF MECHANICAL ENGINEERING
CENTER FOR BIOENGINEERING
UNIVERSITY OF COLORADO SCHOOL OF MEDICINE
AURORA, CO 80045

GRITSENKO, VALERIYA, PHD *
ASSOCIATE PROFESSOR
DEPARTMENT OF HUMAN PERFORMANCE AND APPLIED EXERCISE SCIENCE
WEST VIRGINIA UNIVERSITY
MORGANTOWN, WV 26506

HAMMOND, JASON, MD *
ATTENDING PHYSICIAN
SPORTS MEDICINE
MEDSTAR ORTHOPAEDIC INSTITUTE
BALTIMORE, MD 21218

HORNBY, THOMAS GEORGE, PHD, PT *
PROFESSOR
DEPARTMENT OF PHYSICAL MEDICINE AND REHABILITATION
SCHOOL OF MEDICINE
INDIANA UNIVERSITY
INDIANAPOLIS, IN 46254

HUANG, HELEN J, PHD *
ASSOCIATE PROFESSOR
DEPARTMENT OF MECHANICAL AND AEROSPACE ENGINEERING
UNIVERSITY OF CENTRAL FLORIDA
ORLANDO, FL 32816

JAIN, NITIN B, MD
PROFESSOR
DEPARTMENT OF PHYSICAL MEDICINE AND REHABILITATION
POPULATION AND DATA SCIENCES, ORTHOPAEDIC SURGERY
SCHOOL OF MEDICINE
UNIVERSITY OF TEXAS SOUTHWESTERN MEDICAL CENTER
DALLAS, TX 75390

KESAR, TRISHA, PHD
ASSOCIATE PROFESSOR
DIVISION OF PHYSICAL THERAPY
DEPARTMENT OF REHABILITATION MEDICINE
EMORY UNIVERSITY SCHOOL OF MEDICINE
ATLANTA, GA 30322

KIJOWSKI, RICHARD, MD
PROFESSOR
DEPARTMENT OF RADIOLOGY
NEW YORK UNIVERSITY
NEW YORK, NY 10016

KLINE, PAUL W., PHD, DPT *
ASSISTANT PROFESSOR OF PHYSICAL THERAPY
DEPARTMENT OF PHYSICAL THERAPY
ONE UNIVERSITY PARKWAY
HIGH POINT UNIVERSITY
HIGH POINT, NC 27268

MANCINI, MARTINA, ENGD *
ASSOCIATE PROFESSOR
DEPARTMENT OF NEUROLOGY
OREGON HEALTH AND SCIENCE UNIVERSITY
PORTLAND, OR 97239

MOORE, RENEE H, PHD
DIRECTOR AND RESEARCH PROFESSOR OF BIOSTATISTICS COLLABORATION CORE
DEPARTMENT OF EPIDEMIOLOGY AND BIOSTATISTICS
DORNSIFE SCHOOL OF PUBLIC HEALTH
DREXEL UNIVERSITY
PHILADELPHIA, PA 19104

MURRAY, WENDY M, PHD *
PROFESSOR
DEPARTMENT OF BIOMEDICAL ENGINEERING
PHYSICAL MEDICINE AND REHABILITATION
NORTHWESTERN UNIVERSITY
EVANSTON, IL 60208

NYMAN, JEFFRY STEPHEN, PHD *
ASSOCIATE PROFESSOR
DEPARTMENT OF ORTHOPAEDIC SURGERY
VANDERBILT UNIVERSITY MEDICAL CENTER
NASHVILLE, TN 37232

PLUMMER, PRUDENCE, PHD, PT *
PROFESSOR
DEPARTMENT OF PHYSICAL THERAPY
MGH INSTITUTE OF HEALTH PROFESSIONS
BOSTON, MA 02129

QUEEN, ROBIN M, PHD
PROFESSOR
DEPARTMENT OF BIOMEDICAL ENGINEERING AND MECHANICS
COLLEGE OF ENGINEERING
VIRGINIA TECH
BLACKSBURG, VA 24061

SAUL, KATHERINE, PHD *
PROFESSOR
DEPARTMENT OF MECHANICAL AND AEROSPACE ENGINEERING
NORTH CAROLINA STATE UNIVERSITY
RALEIGH, NC 27695

SLAVENS, BROOKE A, PHD
ASSOCIATE PROFESSOR
DEPARTMENT OF MECHANICAL ENGINEERING
COLLEGE OF ENGINEERING AND APPLIED SCIENCE
UNIVERSITY OF WISCONSIN-MILWAUKEE
MILWAUKEE, WI 53201

STEELE, KATHERINE M, PHD
ASSOCIATE PROFESSOR
DEPARTMENT OF MECHANICAL ENGINEERING
COLLEGE OF ENGINEERING
UNIVERSITY OF WASHINGTON
SEATTLE, WA 98195

TROY, KAREN L, PHD *
PROFESSOR
BIOMEDICAL ENGINEERING
WORCESTER POLYTECHNIC INSTITUTE
WORCESTER, MA 01609

SCIENTIFIC REVIEW OFFICER

LOVERING, RICHARD MICHAEL, PHD
SCIENTIFIC REVIEW OFFICER
CENTER FOR SCIENTIFIC REVIEW
NATIONAL INSTITUTES OF HEALTH
BETHESDA, MD 20892

* Temporary Member. For grant applications, temporary members may participate in the entire meeting or may review only selected applications as needed.

Consultants are required to absent themselves from the room during the review of any application if their presence would constitute or appear to constitute a conflict of interest.
